# Flavonoid Biosynthesis Genes Putatively Identified in the Aromatic Plant *Polygonum minus* via Expressed Sequences Tag (EST) Analysis

**DOI:** 10.3390/ijms13032692

**Published:** 2012-02-28

**Authors:** Nur Diyana Roslan, Jastina Mat Yusop, Syarul Nataqain Baharum, Roohaida Othman, Zeti-Azura Mohamed-Hussein, Ismanizan Ismail, Normah Mohd Noor, Zamri Zainal

**Affiliations:** 1 Institute of Systems Biology (INBIOSIS), Universiti Kebangsaan Malaysia, 43600 Bangi Selangor, Malaysia; E-Mails: adiyana112@yahoo.com (N.D.R.); jastina@ukm.my (J.M.Y.); nataqain@ukm.my (S.N.B.); roohaida@ukm.my (R.O.); zeti@ukm.my (Z.-A.M.-H.); maniz@ukm.my (I.I.); normah@ukm.my (N.M.N.); 2 School of Biosciences and Biotechnology, Faculty of Science and Technology, Universiti Kebangsaan Malaysia,43600 Bangi Selangor, Malaysia

**Keywords:** cDNA library, expressed sequence tags, flavonoid biosynthesis, *Polygonum minus*, quantitative real-time PCR

## Abstract

*P. minus* is an aromatic plant, the leaf of which is widely used as a food additive and in the perfume industry. The leaf also accumulates secondary metabolites that act as active ingredients such as flavonoid. Due to limited genomic and transcriptomic data, the biosynthetic pathway of flavonoids is currently unclear. Identification of candidate genes involved in the flavonoid biosynthetic pathway will significantly contribute to understanding the biosynthesis of active compounds. We have constructed a standard cDNA library from *P. minus* leaves, and two normalized full-length enriched cDNA libraries were constructed from stem and root organs in order to create a gene resource for the biosynthesis of secondary metabolites, especially flavonoid biosynthesis. Thus, large-scale sequencing of *P. minus* cDNA libraries identified 4196 expressed sequences tags (ESTs) which were deposited in dbEST in the National Center of Biotechnology Information (NCBI). From the three constructed cDNA libraries, 11 ESTs encoding seven genes were mapped to the flavonoid biosynthetic pathway. Finally, three flavonoid biosynthetic pathway-related ESTs *chalcone synthase*, CHS (JG745304), *flavonol synthase*, FLS (JG705819) and *leucoanthocyanidin dioxygenase*, LDOX (JG745247) were selected for further examination by quantitative RT-PCR (qRT-PCR) in different *P. minus* organs. Expression was detected in leaf, stem and root. Gene expression studies have been initiated in order to better understand the underlying physiological processes.

## 1. Introduction

Aromatic plants are important sources of plant secondary metabolites, which are beneficial for human health in the form of drugs, antioxidants, flavors, fragrances, dyes, insecticides and pheromones [1]. Recent advances in plant genomics, proteomics and metabolomics research have generated knowledge leading to a greater understanding of biosynthetic pathways in aromatic plant [2]. *Polygonum minus* is an aromatic plant for which information regarding the biosynthesis of its secondary metabolites are limited. This plant was selected for study because many of its aromatic compounds have not been identified.

*P. minus*, which is also known as *kesum*, belongs to the Polygonacea family and primarily grows in temperate regions. *P. Minus* is an aromatic plant that contains a variety of valuable aromatic compounds. The leaves are used to relieve indigestion and are also useful in postnatal tonics. Additionally, the essential oil extracted from the leaves of this plant is used as hair shampoo to remove dandruff and also is used in aromatherapy [3]. Various studies have been conducted on this species because of its medicinal value. Most *P. minus* research has focused on the identification of the bio-molecules it produces and their chemical properties. Previous studies has shown that *P. minus* exhibits high antioxidant activity [4], which may be due to its high level of phenolic and flavonoid compounds [5]. Additionally, 10 aldehyde compounds were identified by GC-MS in *P. minus* essential oils [6], and additional compounds were detected when a high-throughput GC-TOF MS analytical tool was used [7].

At present, there is no comprehensive EST database available for the *P. minus* secondary metabolite biosynthetic pathways. Thus, EST analysis could be a valuable approach in the discovery of new genes involved in secondary metabolite production in this non-model plant. EST analysis has been successfully used for the genomic investigation of several important plants, including *Arabidopsis thaliana* [8], rice [9] and medicinal plants. Research by Li *et al*. has identified genes encoding enzymes involved in the biosynthesis of glycyrrhizin from *Glycyrrhiza uralensis* via ESTs [10]. Genes encoding enzymes involved in the biosynthesis of ginsenosides [11] and biosynthesis of withanolides were also recently identified by ESTs [12].

Moreover, genes involved in the flavonoid biosynthesis pathway in young and mature tea leaves have been successfully identified by an EST approach [13]. Flavonoids are an example of aromatic compounds found in *Polygonum spp.* In fact, flavonoids are the most common compounds found in this species, rather than other secondary metabolites such as triterpenoids, anthraquinones, coumarins, phenylpropanoids, lignans, sesquiterpenoids, stilbenoids, and tannins [14]. In recent years, flavonoids have attracted the interest of researchers because they show promise of being powerful antioxidants which can protect the human body from free radicals [15].

Besides, these flavonoids are also important for physiological processes, such as plant growth and development. The first enzyme of the flavonoid pathway, *chalcone synthase* (CHS), catalyses the conversion of *p*-coumaryl coA to naringenin chalcone. Subsequently, *chalcone isomerase* (CHI) catalyses the conversion of chalcone to naringenin, which is then converted to dihydrokaempferol and dihydroquercetin by *flavanone 3-hydroxylase* (F3H) and *flavonoid 3′-hyroxylase* (F3′H), respectively. The pathway can branch into two possible outcomes at this point. At one end, *leucoanthocyanidin dioxygenase* (LDOX) catalyses the conversion of leucoanthocyanidin to anthocyanidin, and at the other, *flavonol synthase* (FLS) converts dihydroflavonol to flavonols (kaempferol and quercetin).

Despite the extensive study of the *Arabidopsis* genes, many non-model organism genomes, such as *P. minus*, remain poorly studied, and little is known about the genes in the *P. minus* flavonoid biosynthetic pathway. Thus, to expand the genomic resources available and to discover genes involved in the *P. minus* flavonoid biosynthetic pathway*,* we constructed a standard cDNA library from a *P. minus* leaf and two normalized full-length enriched cDNA libraries from the stem and root. Following construction of these libraries from the different *P. minus* organs, a transcriptomic dataset was developed to strengthen our metabolomic findings. We identified 4196 unigene transcripts, including transcripts that are possibly associated with transcription control genes, stress-related genes, transporter genes, unknown genes and several candidate genes that have homology with flavonoid genes.

## 2. Results and Discussion

### 2.1. Characterisation of the *P. minus* Standard Library and Normalized Full-Length Enriched cDNA Libraries

To ensure full coverage of the expressed transcripts, three cDNA libraries were generated from different organs. While the standard leaf library for the primary titer was 5 × 10^5^ pfu/μL, the stem and root titer were 1.1 × 10^6^ cfu/μL and 1.4 × 10^6^ cfu/μL, respectively. The cDNA libraries exhibited high efficiency as the recombinant plasmid recovery rate was 95%, with an average insert length of 1 kb. The transcript redundancy was significantly high (30%) in the standard library. However, this was expected because no normalization was performed for this library as it can contain a high frequency of the same expressed genes, thereby affecting the efficiency and EST cost effectiveness [16]. Furthermore, standard cDNA library construction approaches also suffer from several shortcomings, including the fact that the majority of the cDNA clones are not full length and adaptor-mediated cloning results in up to 20% of undesirable ligations by non-mRNA products and inserts [17], making it ineffective for rare transcript discovery. To overcome the aforementioned problems, a new strategy was employed to isolate all classes of transcripts in an equal number. Thus, two normalized full-length enriched cDNA libraries were constructed from the *P. minus* stem and root, and the EST redundancy was low (8.8–9%).

### 2.2. Single-Pass EST Clone Sequencing, Assembly and Annotation

A total of 3260 randomly chosen clones from the standard library were sent for sequencing from the 5′ end. Clones with no insert or sequences shorter than 100 bp were excluded, resulting in 1977 high-quality sequences with a mean length of 630 bp. After selection, the clean sequences were assembled using CAP3 software for clustering, yielding 392 contigs and 922 singletons, which generated 1314 tentatively unigene transcripts. Single-pass sequencing from the 5′ end was conducted for 2016 clones from both the root and stem, which generated two libraries containing 1767 and 1398 high-quality sequences from the root and stem, respectively. The high-quality sequences were then assembled using StackPACK v2.2 into 130 consensus and 1481 singletons, representing 1611 tentatively unigene transcripts for the root. For the stem, there were 1271 tentatively unigene transcripts generated from 92 consensus sequences and 1179 singletons (Table 1). The stem showed fewer high quality sequences compared to root because of possibility that some of the genes may exist in other previously sequenced organisms but have never been expressed or captured for sequencing. These ESTs were deposited in the NCBI under accession numbers JG744053 to JG745366 (leaf), JG700177 to JG701445 (stem), JG 705365 to JG706110 and JG732257 to JG732279 (root).

**Figure 1:**
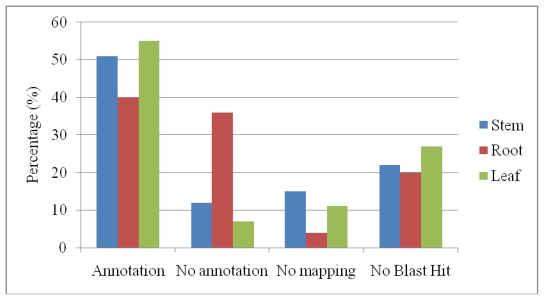
Distribution of annotation, no annotation, no maping and no blast hit for different *P. minus* organs.

Blastx analysis demonstrated that the ESTs could be categorized as annotation, no annotations, no mapping and no blast hits. From (Figure 1) among these sequences, approximately 40–55% and 18–40% showed a significant match with sequences in the non-redundant protein database encoding for “annotations”, “no annotation” and “no mapping” respectively. The root has shown high percentages with “no annotation”. The average length of the ESTs with a poor match for the contigs and singletons in this study were ~400 bp. Therefore, it is most likely that the poor match was not primarily caused by short EST length, but the lack of sequence information in the public protein database. Additionally, 20–27% of the ESTs showed no similarities in any sequence database and was therefore designated as “no blast hits”. High percentages of “no blast hits” were shown by leaf as high sequences with shorter length were found. A decrease in sequence length showed an increased probability of returning no significant matches to the protein database. Sequence lengths of 151 to 250 bp can reach maximum 60% of not getting significant matches [18].

### 2.3. Transcription Profile Analysis

As the libraries were constructed from different organs, the representative ESTs present in those libraries should be varied among the organs, based on their physiological function. A representative gene that can be found in the normalized full-length, enriched root cDNA library is the ABC transporter (Table 2a). The ABC transporter is involved in the transport of secondary metabolite compounds, which are synthesized in the root and transported to the leaves via the xylem [19]. The ABC transporter can be associated with *P. minus* regulation of the transport of secondary metabolite products produced in the root to the leaf. Moreover, it is not only involved, both directly and indirectly, in plant growth and development, but it is also involved in cellular detoxification [20]. The PDR-type ABC transporter (JG732266) also was found in the *P. minus* stem cDNA library, and it has been reported to be involved in the response to pathogens and a broad range of stresses [21] including salinity, cold and heavy metals [22].

The genes most represented in the normalized stem cDNA library were transcription factors (TFs) (Table 2b). TFs are the major proteins in which a cell or organism regulates its gene expression, thus directing temporal and spatial expression for normal development and the responses to physiological or environmental stimuli. Comparative genomic analyses reveal that transcriptional regulator genes are abundant in plants. TF-encoding transcripts such as the GATA TF family (JG700197), the GRAS TF family (JG700265), ARF domain class TFs (JG700211), WRKY TFs (JG701357), MYBR domain class TFs (JG700745) and transcription elongation factor TFs (JG700215) were expressed. All of these types of proteins play an essential role in modulating the transcription rate of specific target genes. For example, in plants, many WRKY proteins are involved in the defense against attack from pathogenic bacteria, fungi, viruses and oomycetes. Additionally, WRKY genes are implicated in responses to the abiotic stresses of wounding and the combination of drought, heat [23] and cold. It is also evident that some TF family members may play important regulatory roles in plant growth and metabolic pathways.

Several transcripts in the EST dataset encoded proteins that may be associated with stress-related genes, such as dehydration-induced proteins, were also found in the normalized full-length enriched stem cDNA library. This protein typically accumulates during low-temperature or water-deficient conditions and is thought to play a role in plant freezing and drought tolerance. The *P. minus* specimens used in this study were collected from a lowland region where it may be assumed that these proteins are not induced due to low temperatures but, rather, that they most likely play a role in drought tolerance in the *P. minus* stem. Another EST for the stress-related gene lipid transfer protein (LTP) (JG745356) was highly expressed in the standard leaf library. LTP plays a role in biological processes that involve plant signaling to enhance pathogen resistance. The LTP isolated from barley and maize leaves showed inhibitory activities towards bacterial pathogens and fungi [24]. Additionally, the LTP gene also reacts with abscisic acid and other agents that promote osmotic stress such as sodium chlorate and mannitol. In this instance, these observed activities suggest that the *P. minus* LTP is involved in processes such as adaptation for the survival of this plant in response to pathogens and stress.

For the standard leaf cDNA library, the most abundantly expressed ESTs were those involved in the photosynthetic system, including 120 chlorophyll a/b binding protein ESTs (JG745226) and 420 ribulose-1,5-biphosphate carboxylase ESTs (JG745178), which were the most highly expressed (Table 2c). These results are similar to that which has been observed in *Stevia reboundiana* [25].

### 2.4. Gene Ontology Annotations

From the EST datasets, functional analysis has revealed that 39.4 to 55.6% of the unigene transcripts are annotated, based on GO conventions. Figure S1 shows the GO mapping for biological processes from different *P. minus* organs. As shown in the bar chart, the most represented biological process for each organ part is “metabolic process”, which includes primary metabolic processes, cellular metabolic processes and macromolecular metabolic processes (Figure S1a). These processes are important for *P. minus* as they are involved in the generation and use of energy in the developmental stages. Other categories associated with biological processes included development processes, biological regulation and response to stimuli. Stimuli can have effects on the overall development and growth of *P. minus* plants. The level of transcripts encoding certain members of the mitogen-activated protein kinase (MAPK) family is increased in response to contact or osmotic stress and wounding [26].

The molecular function category mainly comprised proteins involved in binding, and a large number associated with catalytic activities, such as hydrolase and transferase (Figure S1b), were annotated, suggesting that these *P. minus* libraries may allow for the identification of genes involved in secondary metabolite biosynthesis pathways. For cellular components, most genes were annotated as having intracellular and membrane localization (Figure S1c).

### 2.5. KEGG-Based Biochemical Analysis

As an alternative method in categorizing ESTs by their biochemical function, we assigned the ESTs to metabolic pathways via the *Kyoto Encyclopedia of Genes and Genomes* (KEGG), using enzyme commission (EC) numbers as the basis for assignment. From the 48 unigene transcripts associated with secondary metabolites, we have identified 11 unigene transcripts that correspond to seven enzymes (Table S1), and these 11 unigene transcripts are largely mapped in flavonoid biosynthesis as illustrated in Figure S2. A previous study has shown that *P. minus* has high phenolic compound levels, and it was assumed that the majority of these phenolic compounds were flavonoids.

Furthermore, this finding is in agreement with our preliminary metabolomic data, which indicated that flavonoid compounds are highly abundant in *P. minus* (unpublished data). The majority of flavonoid plant classes include flavonols, flavones, flavanones, flavanols, anthocyanidins, isoflavones, dihydroflavonol and chalcones. Individual plant species can synthesize a variety of flavonoid compounds, which have various functions, such as providing pigmentation to attract pollinators, defending plants against pathogens, acting as signal molecules in plant-microbe interactions, and protecting plants from UV radiation [27]. With respect to the medicinal properties of these compounds, they have been reported to exhibit a wide range of biological effects including antibacterial, antiviral, anti-inflammatory, anti-allergic and vasodilatory actions [28]. The antioxidant activity of plants may be associated with their phenolic compounds. As *P. minus* has a high phenolic compound level, it may have therapeutic benefits associated with its antioxidant properties. Thus, identifying genes involved in flavonoid biosynthetic pathway denotes an important step in expanding our understanding of the secondary metabolites of this plant.

### 2.6. Expression of Flavonoid Biosynthesis-Related Genes

We have employed qRT-PCR to investigate the level of expression of these genes in different organs. Three flavonoid-related genes, *chalcone synthase*, CHS (JG745304), *flavonol synthase*, FLS (JG705819), and *leucoanthocyanidin dioxygenase*, LDOX (JG745247) were amplified by qRT-PCR from different leaf, stem and root tissues using gene-specific primers (Table S2). These three genes were selected because they are involved in the downstream expression of flavonoid biosynthesis pathway. Furthermore, some of the downstream metabolite compounds synthesized by these genes have been found in this plant. CHS was selected as the gene involved in initial step and controls the flavonoid biosynthesis. FLS and LDOX are both synthesized to products that are related to antioxidant compounds such as quercertin, kaempferol and cyanidin. A correlation between transcript expression and metabolites is taken as direct evidence of gene function in reverse genetics. A combination of targeted metabolomic and transcriptomic approaches in distinct cell types and organs may be useful for determining gene-metabolite relationships [29].

**Figure 2:**
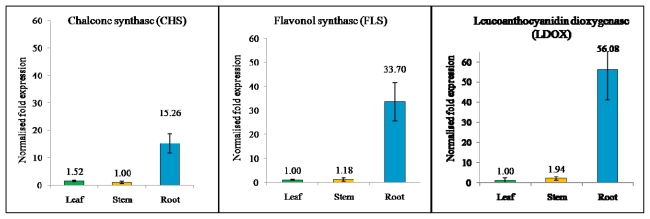
SYBR-Green Real Time PCR analysis of flavonoid-related genes in three different tissues as indicated. (**a**) *Chalcone synthase*, CHS (GenBank: JG745304); (**b**) *Flavonol synthase*, FLS (GenBank: JG705819); and (**c**) *Leucoanthocyanidin dioxygenase*, LDOX (GenBank: JG745247). mRNA levels for each were expressed relative to the amount of *Tubulin* and *β-actin* mRNA. Data are presented as mean ± SE (*n* = 3), in arbitrary units.

Based on the partial sequences obtained from EST data, primer pairs were designed for specifically detecting the expression level of flavonoid-related genes through qRT-PCR. ANOVA analysis showed expression of all the genes in different organs had significant differences with *P* < 0.05. As shown in Figure 2, the expression pattern indicated that these flavonoid-related genes were expressed in every *P. minus* organ with different expression levels. The CHS (2.3.1.74) transcripts were expressed 10-fold higher in the root compared to the leaf and 15-fold compared to stem. CHS has also been found in the pea root [30]. The higher CHS transcript level in roots reflects a higher synthesis of flavonoid derivatives in legume roots, which are secreted into the rhizosphere to induce the Rhizobium nod genes.

Meanwhile, FLS (1.14.11.23) transcripts exhibited the highest expression, with 28-fold higher expression in the root as compared to the stem and 34-fold compared to the leaf. This is probably the FLS involved in modulation auxin transport of root. The expression of FLS can produce quercetin and kaempferol which have been identified as the most active flavonoids acting as regulators for the transport of auxin, and thereby affecting root development in plants [31]. In addition, the flavonol (quercertin and kaempferol) also play important roles in defense activity against pathogens and insect attacks, and also act as key signaling molecules in the formation of nitrogen-fixing root nodules in legumes [32].

Finally, LDOX (1.14.11.19), a late gene 2-oxoglutarate iron-dependent oxygenase that is involved in anthocyanin biosynthesis, catalyses a step in the biosynthesis of the anthocyanin class of flavonoids from colorless leucoanthocyanidins to colored anthocyanidins [33]. Based on qRT-PCR, LDOX showed a 29-fold expression change in the root compared to the stem and 56-fold compared to leaf. The expression was supported by Liu *et al*. (2010) [34] showing that LDOX was expressed highly in root and the product synthesized by LDOX was transferred to another part. In most plant species, anthocyanin production is limited to certain tissues and occurs during specific developmental stages. The visible accumulation of these compounds usually reflects the activity of the biosynthetic enzymes functioning in the pathway [35]. Another possible reason is that anthocyanidins act as determining factors for plant structure pigmentation. The *P. minus* stem and root has a reddish hue compared to the leaf, and this pigmentation describes the LDOX expression pattern.

All flavonoid-related genes gave a distinct pattern in different tissues, indicating that the flavonoid genes may undergo differential regulation. This indication was supported in research by Buer and colleagues that showed flavonoids can translocate to different positions in the plant to regulate physiological processes [36]. The spatial expression characteristics also showed their functional sites and will help us to isolate the full length of these genes. Furthermore we will be able to manipulate these genes and regulate the flavonoid pathways via overexpression or down-regulation of key genes for antioxidant compound.

## 3. Experimental Section

### 3.1. Plant Material and Sample Collection

One standard cDNA library from the leaf and two normalized full-length cDNA enriched libraries from the stem and root were constructed. The plant material for library construction was collected from Ulu Yam and was grown at a Universiti Kebangsaan Malaysia (UKM) plot. All plant organs parts were then harvested and washed under running tap water.

### 3.2. Construction of Normal cDNA Library from *P. minus* Leaf

Construction of a normal *P. minus* cDNA library was performed using total RNA prepared from 5 grams of leaf tissue using the Lopez and Gomez method [37]. The leaf used was excised at the third position node from the top. Due to the high levels of phenolic compounds in *P. minus*, modification to the extraction buffer was done by adding 50% PVP-40. PolyA mRNA was purified using the PolyA Tract mRNA Isolation System (Promega, USA). Double-stranded cDNA was synthesized from 5 μg of mRNA with a ZAP-cDNA synthesis kit (Stratagene). After blunting the termini, the cDNAs were ligated to *Eco*RI adapters, and the *Eco*RI ends were phosphorylated following digestion with *Xho*I. The cDNA was size fractionated into separate fragments of over 500 bp in length in 0.8% agarose gels. The cDNAs were purified and ligated into the Uni-ZAP XR vector, and Gigapack III Gold Packaging Extract (Stratagene) was used to package the ligated products. The library was titred and amplified, and the insertion efficiency was assessed. For DNA sequencing, the library was amplified and the insert DNA was excised *in vivo* from the vector with ExAssist Helper Phage.

### 3.3. Construction of Normalized Full-Length Enriched cDNA Libraries from *P. minus* Root and Stem

The cDNA libraries were constructed by Vertis Biotechnologie, Germany. Full-length enrichment (FLE) was chosen as the cDNA synthesis method. Approximately 10 g of roots and 10 g of stems were ground to form a fine powder in liquid nitrogen using Lopez and Gomez methods. After that, 100 μg of RNA was purified with an RNeasy Plant Mini Kit (Qiagen), and its quantity and quality were checked using gel electrophoresis and Nanodrop. Next, approximately 25 μg of total RNA were sent to Vertis Biotechnologie for cDNA construction in 1/10 volume of 3 M NaOAC, pH 5.2 and 3 volume of 100% ethanol because RNA is stable for some time at an elevated temperature. During cDNA synthesis, oligonucleotide primers were attached to the 5′ and 3′ ends of the cDNA to allow for PCR amplification of the cDNA and directional cloning of the cDNA into the 5′ EcoRI*/*3′ *Bam*HI-sites of pBluescript II SK(+). Normalization by denaturation/re-association and subsequent separation of double and single-stranded DNA using a hydroxyapatite column was performed to equalize transcript abundance. The cDNAs were then separated by agarose gel electrophoresis and size fractionated (>0.5 kb) before cloning and transforming into the *E. coli* strain NEB 10-β.

### 3.4. Single-Pass Sequencing

Bacterial colonies were incubated in 1.2 mL of LB containing 100 μg/μL ampicillin in shaking incubator at 37 °C at 320 rpm for 20 to 24 h. Plasmids were extracted using the Montage Plasmid MiniPrep MILLIPORE kit. RNase A was added in solution 1 to digest contaminating RNA before purifying the plasmids. The samples were then sent for sequencing at the Malaysian Genome Institute (MGI) by using T7 primers at the 5′-end.

### 3.5. EST Clustering and Annotations

Filtering was performed using the Malaysian Genome Institute (MGI) pipeline. Raw chromatograms were base-called using Phred 20 with an error probability cut-off of 0.01. Vector sequences were trimmed using Cross-match, and clean sequences were assembled and clustered with StackPACK v2.2. In this pipeline, edited files were assembled into high sequence identity groups using the D2_cluster (clusters), phrap (contigs) and craw (consensus sequences) algorithms. All unigene transcripts were compared with the non-redundant (nr) database. Gene ontology (GO) annotations were performed using Blast2Go. The unigene transcripts were imported into Blast2Go and BlastP with a minimum *E*-value of 10^−3^ prior to mapping and annotation into GO terms. The unigene transcripts were annotated and mapped according to the Kyoto Encyclopaedia of Genes and Genomes (KEGG) and orthology (KO) by the KEGG Automatic Annotations server (KAAS).

### 3.6. qRT-PCR of Selected Flavonoid Related Genes

Total RNA was extracted from the *P. minus* leaf, stem and root according to a modified Lopez and Gomez (1992) method. Prior to reverse transcription, 6 μg of RNA samples were treated with DNase using the DNaseI kit (Promega, USA) in accordance with the manufacturer’s protocol. DNase-treated RNA (2 μg) was reverse transcribed with Maxima Enzyme Mix (Fermentas, Europe) in a volume of 20 μL. Three unigene transcripts from the flavonoid biosynthesis pathway were selected from the EST dataset for qRT-PCR analysis using the primers shown in Table S2. qRT-PCR was performed in an optical 96-well plate using a Biorad iQ5 (Biorad) and universal cycling conditions (3 min at 95 °C, 40 cycles of 10 s at 95 °C and 30 s at 56 °C) followed by the generation of a melting curve to check the amplification specificity. The reaction mixture contained iQ5 SYBR Green Master Mix (Biorad), 100 μM of the specific primers and 100 ng of cDNA in 15 μL reactions. Primer efficiencies and standard deviations were calculated on a standard curve generated using different concentrations of cDNA for 5 dilution points in triplicate. *Tubulin* and *β-actin* were selected as reference genes.

### 3.7. Statistical Analysis

Data were expressed as mean ± SE. Two-way ANOVA and Tukey’s tests (Minitab Inc., USA) were performed to assess the statistical significance between organs. Statistical significance was set at *P* < 0.05.

## 4. Conclusions

In conclusion, three libraries were constructed from the leaf, stem and root of *P. minus* and provided an efficient resource for gene discovery. In this study, we report the first EST constructed from *P. minus* and based on the EST analysis. We have identified several unigenes transcripts involved in the flavonoid biosynthesis pathway and developmental regulation in *P. minus.* This genomics approach will accelerate gene discovery and provide promising targets for genetic engineering of antioxidant production.

## Supplementary Information



## Figures and Tables

**Table 1:** Statistics for ESTs found in different *P. minus* organs.

Features	Standard leaf library	Normalized stem library	Normalized root library
Total number of clones sequenced	3260	2016	2016
Number of high-quality sequences	1977	1398	1767
Average length of high-quality ESTs (bp)	630 bp	600 bp	600 bp
Number of consensus/contigs	392 contigs	92 consensus	130 consensus
Number of singletons	922	1179	1481
Number of unigene transcripts (UT)	1314 a	1271 a	1611 a
Redundant ESTs	663 (33.5) b	127 (9) b	156 (8.8) b
Functional analysis:			
UT with GO match	731 (55.6) c	657 (51.6) c	636 (39.4) c
Molecular function	1392	1036	832
Biological process	1779	1482	1321
Cellular component	816	692	918
UTs without GO match	583 (44.4) c	614 (48.4) c	975 (60.6) c

a Number of unigene transcripts is the total of consensus sequences and singletons;

b Percentage calculated from a total of quality sequences;

c Percentage calculated from a total of UTs.

**Table 2:** (**a**) Representative ESTs found in the *P. minus* normalized and full-length enriched root library; (**b**) Representative ESTs found in the *P. minus* normalized and full-length enriched stem library; (**c**) The most abundant ESTs in the *P. minus* leaf.

(a)					

UT ID	EST Count	Description	Organism Source	Identity	Accession
root_cn10	2	Multidrug/pheromone exporter, MDR family, ABC transporter family	*Populus trichocarpa*	50	JG732266
root_cn11	2	Hypothetical protein LOC100280328 (*putative ABC transporter)*	*Zea mays*	86	JG732267
root_cn8	2	Os03g0641200 (*Amino acid/polyamine transporter I family protein*)	*Oryza sativa Japonica Group*	66	JG732264
root_cn1	2	Dipeptidyl-peptidase, putative	*Ricinus communis*	84	JG732257
root_cn2	2	Calcium-binding EF hand family protein	*Arabidopsis thaliana*	49	JG732258
root_cn3	2	Cation-transporting ATPase plant, putative	*Ricinus communis*	78	JG732259
root_cn4	2	Unnamed protein product	*Vitis vinifera*	84	JG732260
root_cn7	2	Ormdl, putative	*Ricinus communis*	89	JG732263
root_cn12	2	Small glutamine-rich tetratricopeptide repeat-containing protein A, putative (*co-chaperon)*	*Ricinus communis*	70	JG732268
root_cn14	2	Predicted protein	*Populus trichocarpa*	48	JG732270

(b)					

UT ID	EST Count	Description	Organism Source	Identity	Accession
stem_cn65	4	Photosystem II protein K	*-*	62	JG700241
stem_cn33	3	Predicted protein	*Populus trichocarpa*	99	JG700209
stem_cn42	3	Predicted: similar to small nuclear ribonucleoprotein E	*Vitis vinifera*	94	JG700218
stem_cn83	3	Os12g0207300 (*Clathrin coat assembly protein AP17*)	*Oryza sativa* Japonica Group	98	JG700257
stem_cn39	2	Transcription elongation factor, putative (TF)	*Ricinus communis*	91	JG700215
stem_cn21	2	GATA transcription factor, putative (TF)	*Ricinus communis*	81	JG700197
stem_cn91	2	GRAS family transcription factor (TF)	*Populus trichocarpa*	67	JG700265
stem_cn35	2	ARF domain class transcription factor (TF)	*Malus x domestica*	48	JG700211
stem_cn1	2	Dehydration-induced protein	*Aegiceras corniculatum*	50	JG700177
S005.A07	1	WRKY Protein (TF)	*Rheum australe*	43	JG701357
S017.A08	1	MYBR domain class transcription factor (TF)	*Malus x domestica*	71	JG700745

(c)					

UT ID	EST count	Description	Organism Source	Identity	Accession
leaf_cn134	420	Putative ribulose-1,5-biphosphate carboxylase small subunit precursor	*Rumex obtusifolius*	85	JG745178
leaf_cn312	140	Lipid transfer protein	*Dianthus caryophyllus*	52	JG745356
leaf_cn185	110	Chlorophyll a-b binding protein 3C-like	*Solanum tuberosum*	94	JG745226
leaf_cn4	99	Hairpin-induced protein	*Rheum australe*	69	JG745048
leaf_cn278	63	S-adenosylmethionine synthase	*-*	93	JG745322
leaf_cn184	60	AF220527_1 chlorophyll a/b binding protein precursor	*Euphorbia esula*	91	JG745229
leaf_cn20	55	14 kDa proline-rich protein DC2.15 precursor, putative	*Ricinus communis*	77	JG745064
