# Supplementary material for: Flavonoid Biosynthesis Genes Putatively Identified in the Aromatic Plant Polygonum minus via Expressed Sequences Tag (EST) Analysis
Source: Int J Mol Sci. 2012 Feb 28;13(3):2692–706. doi: 10.3390/ijms13032692 (PMC3317681; doi:10.3390/ijms13032692)
Supplement: Supplementary file 1 [file ijms-13-02692-s001.pdf]

Supplementary Information

1. Flavonoid Associated Gene in *P.minus*

Table S1. Of 48 unique transcripts related to secondary metabolite, 11 unique transcripts flavonoid associated gene was found in *P.minus*

Gene	EC Number	Similarity	*E*-value	Species	EST
		**Flavonoid**			
naringenin-chalcone synthase	2.3.1.74	96	2e-80	*Fallopia multiflora*	321541_P61_D12_M13F-pUC-40<br>cn260
leucoanthocyanidin dioxygenase	1.14.11.19	95<br>95	1e-04<br>7e-52	*Phytolacca americana*<br>*Fagopyrum esculentum*	cn202<br>cn203
shikimate O-hydroxycinnamoyltransferase	2.3.1.133	79	8e-56	*Ricinus communis*	R008.D07.ab1
flavanone 3-dioxygenase	1.14.11.9	75	2e-26	*Camelia sinensis*	R005.C12.ab1
leucoanthocyanidin reductase	1.17.1.3	74	4e-74	*Ricinus communis*	R003.E05.ab1
caffeoyl-CoA O-methyltransferase	2.1.1.104	87	1e-38	*Arabidopsis thaliana*	R009.E03.ab1
flavonol synthase	1.14.11.23	75	2e-26	*Camelia sinensis*	R005.C12.ab1
caffeoyl-CoA O-methyltransferase	2.1.1.104	90<br>68	6e-70<br>4e-49	*Arabidopsis thaliana*<br>*Carthamus tinctorius*	S013.F07<br>S018.D09
leucoanthocyanidin dioxygenase	1.14.11.19	90	3e-24	*Phytolacca americana*	S001.A10

Table S2. Primers for genes of interest and two housekeeping genes with the length of the amplified product.

Genes	Accession	EST	Primer sequences	Length	Size amplicon (bp)
**Flavonoid Genes**					
Flavonol synthase (FLS)	JG705819	R005.C12	*pm*FLS fwd : 5' AGGAGTCTGGTGAACAAGGAG 3'	22	128
			*pm*FLS rev : 5' TTGGTTGCGTATGCTGAAGG 3'	21	
Naringenin chalcone synthase (CHS)	JG745304	cn260	*pm*CHS fwd : 5' ACTTGCCTCGTCGCCTTGA 3'	19	116
			*pm*CHS rev : 5' CTCCCTCTTCTGGATTGC 3'	18	
Leucoanthocyanidin dioxygenase (LDOX)	JG745247	cn203	*pm*LDOX fwd : 5' TGCTGAGGATGGTGATGGTGTC 3'	22	143
			*pm*LDOX rev : 5' ACGGACGTGAGTGCCTTGAC 3'	20	
**Internal control**					
β-actin	JG744126	292178_P55_F5_M13F	*pm*B-actin fwd : 5’ AGCCGCCACTGAGCACAAT 3’	19	131
			*pm*B-actin rev : 5’ GGTCCTCTTCCAGACTTCCATGA 3’	23	
Tubulin			*pm*TUB fwd: 5’ TACCAGCCACCAACCGTAGTCC 3’	22	150
			*pm*TUB rev: 5’ CCAACCTCCTCGTAGTCTTTCTCAA 3’	25	

2. Gene Ontology Anotation

Figure S1. Distribution of unigenes with GO terms associated with biological processes, molecular functions and cellular components. The grouped *P. minus* EST sequences were assigned GO terms according to Blast2Go software. (**a**) Biological process; (**b**) Cellular component; (**c**) Molecular function.

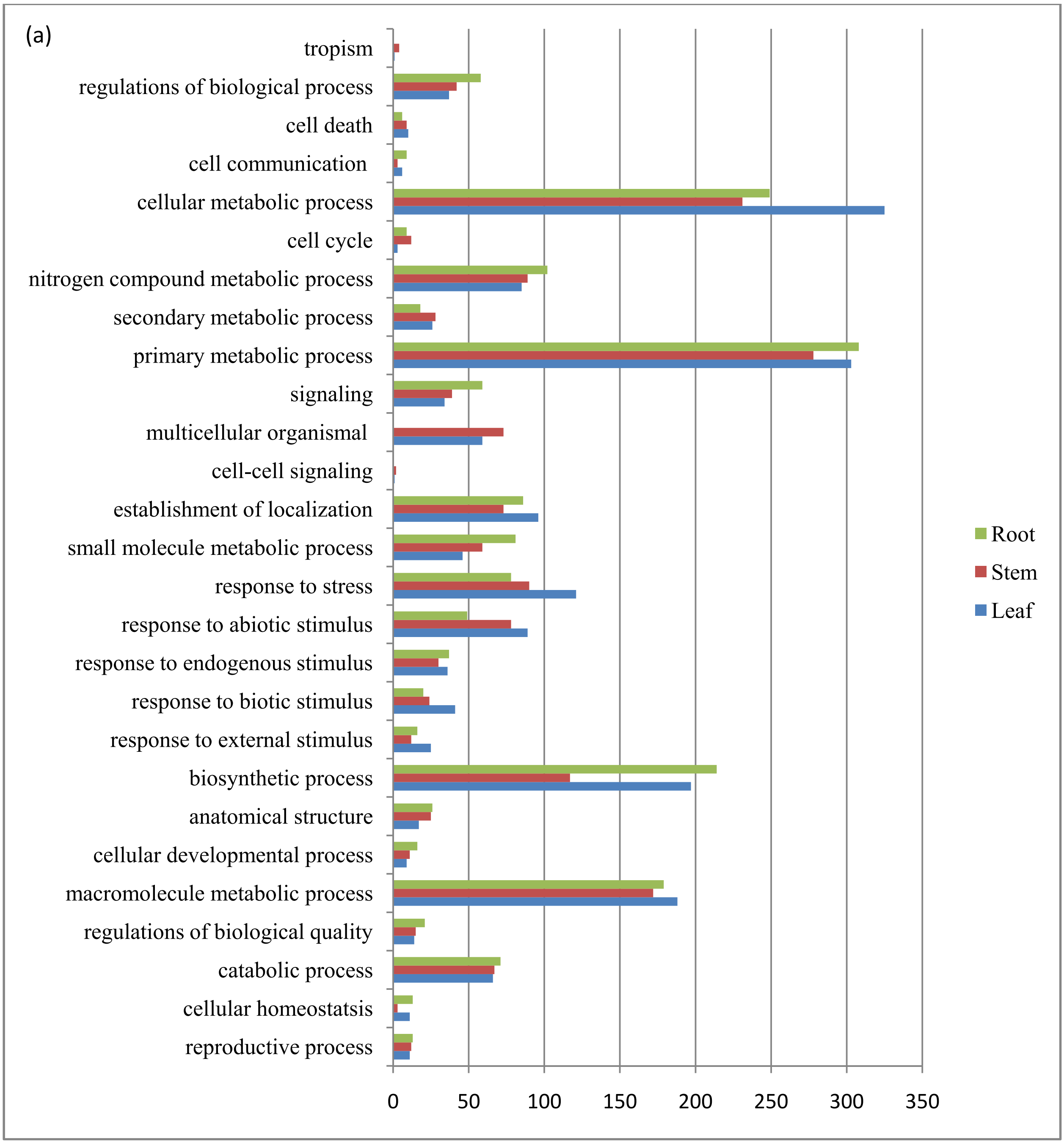

Figure S1. *Cont.*

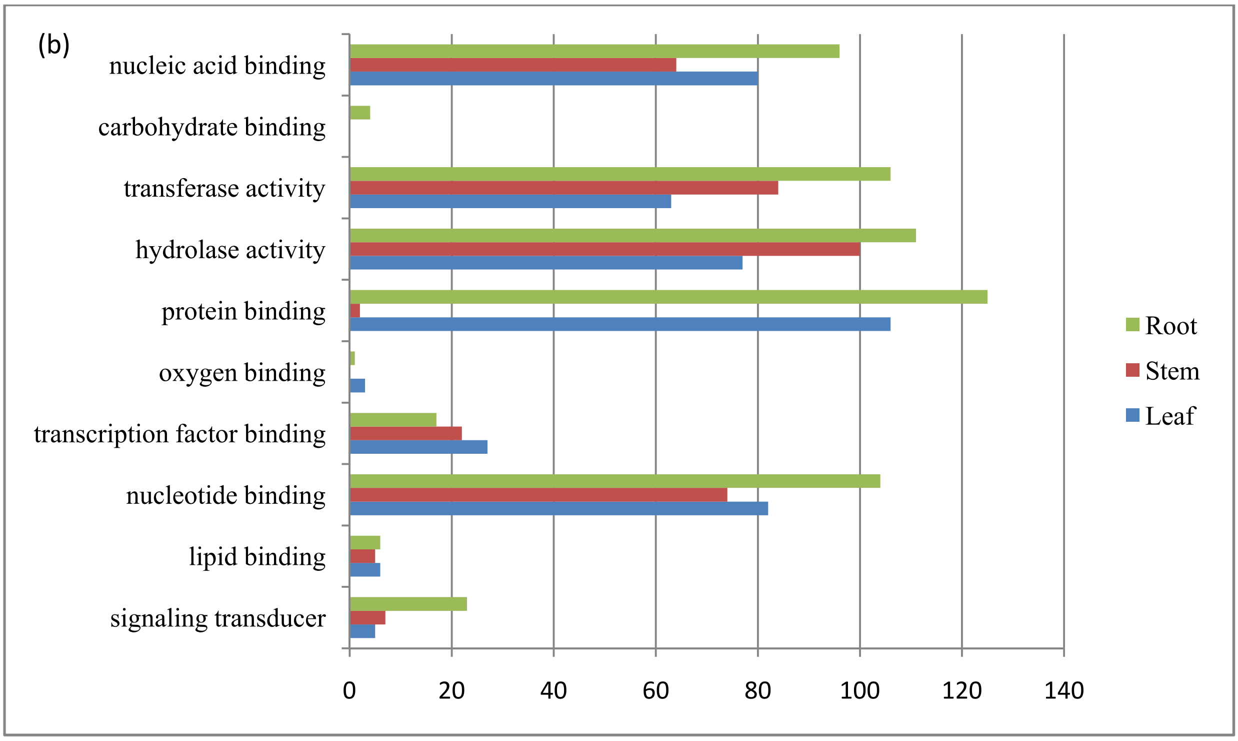

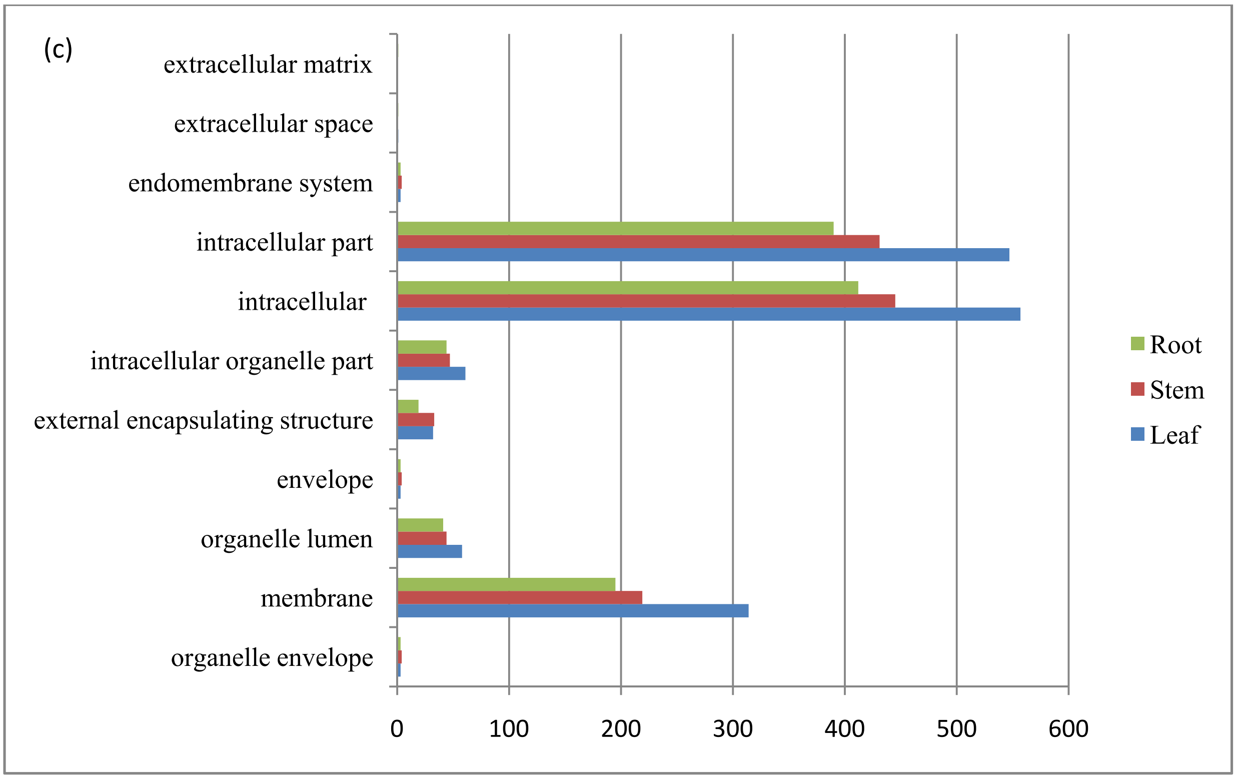

Figure S2. Flavonoid Biosynthesis Pathway in *P.minus*. This pathway was constructed with integrated of EST data (Blue)and metabolomic data (Red).

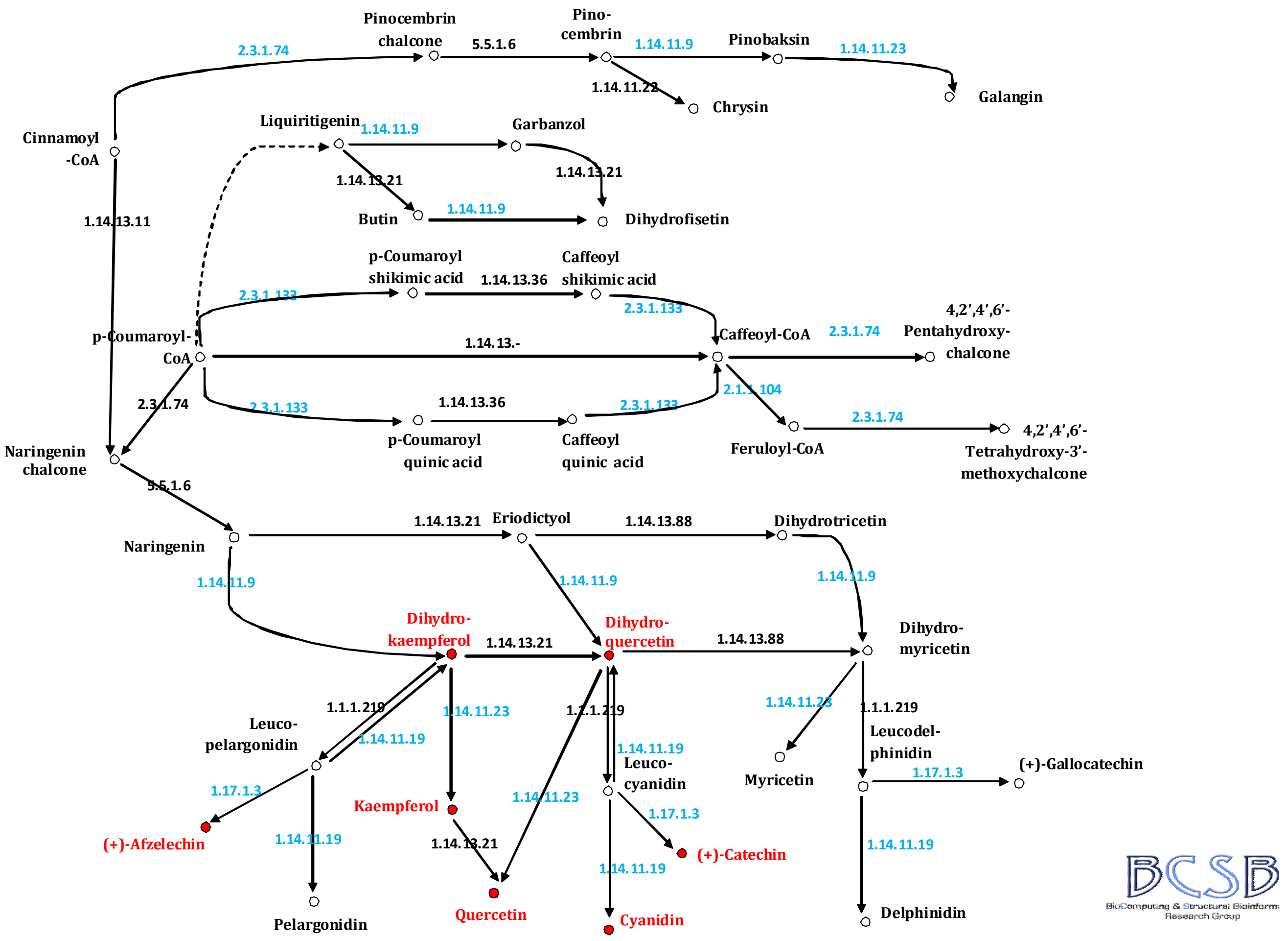

© 2012 by the authors; licensee MDPI, Basel, Switzerland. This article is an open access article distributed under the terms and conditions of the Creative Commons Attribution license (http://creativecommons.org/licenses/by/3.0/).
